# Supplementary material for: Evaluation of Nasal Microbial Communities of Beef Calves During Pre-Weaning Outbreak of Bovine Respiratory Disease
Source: Animals (Basel). 2025 Oct 7;15(19):2914. doi: 10.3390/ani15192914 (PMC12523534; doi:10.3390/ani15192914)
Supplement: Supplementary file 1 [file animals-15-02914-s001.zip › animals-3873933-supplementary.pdf]

Supplemental Table 1. Forward primers with indexes used for PCR amplification of the 16S rRNA gene

Primer Name	Sequence (5'-3')
27F	AGAGTTTGATCCTGGCTCAG
27F_TruSeq1_Diverse	CAAGCAGAAGACGGCATACGAGAT**GTCAAA**GTGACTGGAGTTCAGACGTGTGCTCTTCCGATCTATAGAGTTTGATCCTGGCTCAG
27F_TruSeq2_Diverse	CAAGCAGAAGACGGCATACGAGAT**GTGACA**GTGACTGGAGTTCAGACGTGTGCTCTTCCGATCTCGAGAGTTTGATCCTGGCTCAG
27F_TruSeq3_Diverse	CAAGCAGAAGACGGCATACGAGAT**CATCAT**GTGACTGGAGTTCAGACGTGTGCTCTTCCGATCTGATCAGAGTTTGATCCTGGCTCAG
27F_TruSeq4_Diverse	CAAGCAGAAGACGGCATACGAGAT**GCCGTT**GTGACTGGAGTTCAGACGTGTGCTCTTCCGATCTGCTAAGAGTTTGATCCTGGCTCAG
27F_TruSeq5_Diverse	CAAGCAGAAGACGGCATACGAGAT**CGGCTT**GTGACTGGAGTTCAGACGTGTGCTCTTCCGATCTACTGACAGAGTTTGATCCTGGCTCAG
27F_TruSeq6_Diverse	CAAGCAGAAGACGGCATACGAGAT**GTTCCG**GTGACTGGAGTTCAGACGTGTGCTCTTCCGATCTTGACCAAGAGTTTGATCCTGGCTCAG
27F_TruSeq7_Diverse	CAAGCAGAAGACGGCATACGAGAT**GTAGAT**GTGACTGGAGTTCAGACGTGTGCTCTTCCGATCTGTTACCAGAGTTTGATCCTGGCTCAG
27F_TruSeq8_Diverse	CAAGCAGAAGACGGCATACGAGAT**CCTTCT**GTGACTGGAGTTCAGACGTGTGCTCTTCCGATCTACCGTTAGAGTTTGATCCTGGCTCAG
27F_TruSeq9_Diverse	CAAGCAGAAGACGGCATACGAGAT**TATGCT**GTGACTGGAGTTCAGACGTGTGCTCTTCCGATCTATCGAGAGTTTGATCCTGGCTCAG
27F_TruSeq10_Diverse	CAAGCAGAAGACGGCATACGAGAT**CAGGTA**GTGACTGGAGTTCAGACGTGTGCTCTTCCGATCTCGATAGAGTTTGATCCTGGCTCAG
27F_TruSeq11_Diverse	CAAGCAGAAGACGGCATACGAGAT**GTAGAC**GTGACTGGAGTTCAGACGTGTGCTCTTCCGATCTGACTAGAGTTTGATCCTGGCTCAG
27F_TruSeq12_Diverse	CAAGCAGAAGACGGCATACGAGAT**GACGTT**GTGACTGGAGTTCAGACGTGTGCTCTTCCGATCTCTGAAGAGTTTGATCCTGGCTCAG
27F_TruSeq13_Diverse	CAAGCAGAAGACGGCATACGAGAT**AGCATG**GTGACTGGAGTTCAGACGTGTGCTCTTCCGATCTAGCTCTAGAGTTTGATCCTGGCTCAG
27F_TruSeq14_Diverse	CAAGCAGAAGACGGCATACGAGAT**ATTCGA**GTGACTGGAGTTCAGACGTGTGCTCTTCCGATCTCAGCTAAGAGTTTGATCCTGGCTCAG
27F_TruSeq15_Diverse	CAAGCAGAAGACGGCATACGAGAT**CATCGT**GTGACTGGAGTTCAGACGTGTGCTCTTCCGATCTCAGTAGAGTTTGATCCTGGCTCAG
27F_TruSeq16_Diverse	CAAGCAGAAGACGGCATACGAGAT**GATCGT**GTGACTGGAGTTCAGACGTGTGCTCTTCCGATCTTGACAGAGTTTGATCCTGGCTCAG
27F_TruSeq17_Diverse	CAAGCAGAAGACGGCATACGAGAT**CACTGT**GTGACTGGAGTTCAGACGTGTGCTCTTCCGATCTATAGAGTTTGATCCTGGCTCAG
27F_TruSeq18_Diverse	CAAGCAGAAGACGGCATACGAGAT**CAGTTC**GTGACTGGAGTTCAGACGTGTGCTCTTCCGATCTGTTACCAGAGTTTGATCCTGGCTCAG
27F_TruSeq19_Diverse	CAAGCAGAAGACGGCATACGAGAT**ACAGAT**GTGACTGGAGTTCAGACGTGTGCTCTTCCGATCTGATCAGAGTTTGATCCTGGCTCAG
27F_TruSeq20_Diverse	CAAGCAGAAGACGGCATACGAGAT**AGCGTT**GTGACTGGAGTTCAGACGTGTGCTCTTCCGATCTGCTATTAGAGTTTGATCCTGGCTCAG
27F_TruSeq21_Diverse	CAAGCAGAAGACGGCATACGAGAT**ATAGCT**GTGACTGGAGTTCAGACGTGTGCTCTTCCGATCTCGGAGTAGAGTTTGATCCTGGCTCAG
27F_TruSeq22_Diverse	CAAGCAGAAGACGGCATACGAGAT**CCGTAG**GTGACTGGAGTTCAGACGTGTGCTCTTCCGATCTTCTACCAGAGTTTGATCCTGGCTCAG
27F_TruSeq23_Diverse	CAAGCAGAAGACGGCATACGAGAT**CGCTAG**GTGACTGGAGTTCAGACGTGTGCTCTTCCGATCTACGGATAGAGTTTGATCCTGGCTCAG
27F_TruSeq24_Diverse	CAAGCAGAAGACGGCATACGAGAT**CTATGT**GTGACTGGAGTTCAGACGTGTGCTCTTCCGATCTTCACGAAGAGTTTGATCCTGGCTCAG
27F_TruSeq25_Diverse	CAAGCAGAAGACGGCATACGAGAT**GACATA**GTGACTGGAGTTCAGACGTGTGCTCTTCCGATCTATCCTTAGAGTTTGATCCTGGCTCAG
27F_TruSeq26_Diverse	CAAGCAGAAGACGGCATACGAGAT**GCACTT**GTGACTGGAGTTCAGACGTGTGCTCTTCCGATCTATCGCTAGAGTTTGATCCTGGCTCAG
27F_TruSeq27_Diverse	CAAGCAGAAGACGGCATACGAGAT**GCATTG**GTGACTGGAGTTCAGACGTGTGCTCTTCCGATCTTACGATAGAGTTTGATCCTGGCTCAG
27F_TruSeq28_Diverse	CAAGCAGAAGACGGCATACGAGAT**GCTCTA**GTGACTGGAGTTCAGACGTGTGCTCTTCCGATCTGACTCGAGAGTTTGATCCTGGCTCAG
27F_TruSeq29_Diverse	CAAGCAGAAGACGGCATACGAGAT**GGCACT**GTGACTGGAGTTCAGACGTGTGCTCTTCCGATCTCTGAGGAGAGTTTGATCCTGGCTCAG
27F_TruSeq30_Diverse	CAAGCAGAAGACGGCATACGAGAT**GTACGA**GTGACTGGAGTTCAGACGTGTGCTCTTCCGATCTTGCTATAGAGTTTGATCCTGGCTCAG
27F_TruSeq31_Diverse	CAAGCAGAAGACGGCATACGAGAT**GTTCGC**GTGACTGGAGTTCAGACGTGTGCTCTTCCGATCTAAGCTTAGAGTTTGATCCTGGCTCAG
27F_TruSeq32_Diverse	CAAGCAGAAGACGGCATACGAGAT**TACATT**GTGACTGGAGTTCAGACGTGTGCTCTTCCGATCTCTAGATAGAGTTTGATCCTGGCTCAG
27F_TruSeq33_Diverse	CAAGCAGAAGACGGCATACGAGAT**TCACCT**GTGACTGGAGTTCAGACGTGTGCTCTTCCGATCTATGACTAGAGTTTGATCCTGGCTCAG
27F_TruSeq34_Diverse	CAAGCAGAAGACGGCATACGAGAT**TCGACA**GTGACTGGAGTTCAGACGTGTGCTCTTCCGATCTGTACTGAGAGTTTGATCCTGGCTCAG
27F_TruSeq35_Diverse	CAAGCAGAAGACGGCATACGAGAT**TCTAGT**GTGACTGGAGTTCAGACGTGTGCTCTTCCGATCTATCTCGAGAGTTTGATCCTGGCTCAG
27F_TruSeq36_Diverse	CAAGCAGAAGACGGCATACGAGAT**TGCTGT**GTGACTGGAGTTCAGACGTGTGCTCTTCCGATCTCTGCGAAGAGTTTGATCCTGGCTCAG
27F_TruSeq37_Diverse	CAAGCAGAAGACGGCATACGAGAT**TTCACG**GTGACTGGAGTTCAGACGTGTGCTCTTCCGATCTACAGCTAGAGTTTGATCCTGGCTCAG

27F_TruSeq38_Diverse	CAAGCAGAAGACGGCATACGAGAT**TGACTT**GTGACTGGAGTTCAGACGTGTGCTCTTCCGATCTTTGACCAGAGTTTGATCCTGGCTCAG
519R_A - TruSeqUni	AATGATACGGCGACCACCGAGATCTACACTCTTTCCCTACACGACGCTCTTCCGATCTGTATTACCGCGGCTGCTG

The underlined portion is the Universal 16S Illumina adapter sequence.

The bold bases are the index

Supplemental Table 2. Alpha diversity at genus level of taxonomy. Alpha diversity metrics using a linear mixed model between groups of calves that experienced a BRDC outbreak (Outbreak Group), at the time of the outbreak and approximately 4 weeks post treatment (Timepoint)

Term	Df	Sum Sq	F-value	*P*-value
Outbreak Group	1	149.953	38.717	<0.0001
Timepoint	1	213.120	55.026	<0.0001
Group: Timepoint	1	0.064	0.016	0.898

Variance Components	Estimate	Standard Error
AnimalID	0.636	0.224
Error	3.873	0.191

Supplemental Table 3. Beta diversity at genus level of taxonomy. Beta diversity metrics using permutational multivariate analysis of variance (PERMANOVA) between groups at the time of a BRDC outbreak (MT1, mass treated BRDC outbreak group 1; MT2, mass treated BRDC outbreak group 2) and approximately 4 weeks post-treatment (PT1, BRDC outbreak group 1; PT2, BRDC outbreak group 2).

Term	Df	Sum Sq	R^2	F-value	*P*-value
Treatment	1	3.9945	0.1285	15.860481	<0.001
Animal	54	13.5999	0.43749	1.4679	<0.001
Timepoint	1	3.7388	0.12027	21.7911	<0.001
Treatment:Timepoint	1	0.8315	0.02675	4.8465	0.003
Residual	52	8.9218	0.287		
Total	109	31.0864	1		

Supplemental Table 4. Average abundance and percent positivity of respiratory pathogens in calves. Average Ct value and percentage of samples positive for *Mycoplasma* species *M. bovis*, *M. dispar,* and *M. bovirhinis* for calves.

	Time Point	***M. bovis*** **Average Ct**	***M. dispar*** **Average Ct**	***M. bovirhinis*** **Average Ct**
MT1	Outbreak	29.95	30.03	22.37
MT2	Outbreak	29.99	30.35	19.32
MT1	Post-treatment	32.86	35.88	29.43
MT2	Post-treatment	33.86	30.43	24.65

	Time Point	***M. bovis*** **% Positive**	***M. dispar*** **% Positive**	***M. bovirhinis*** **% Positive**
MT1	Outbreak	95.00	95.00	100.00
MT2	Outbreak	100	94.44	100.00
MT1	Post-treatment	90.00	50.00	65.00
MT2	Post-treatment	83.33	94.44	88.89
